# Epidemiology of infertility and characteristics of infertile couples requesting assisted reproduction in a low-resource setting in Africa, Sudan

**DOI:** 10.1186/s40738-019-0060-1

**Published:** 2019-07-18

**Authors:** Osama G. Elhussein, Mohamed A. Ahmed, Suliman O. Suliman, leena I. Yahya, Ishag Adam

**Affiliations:** 1 Faculty of Medicine, University of Khartoum, PO Box 102, Khartoum, Sudan; 2 Department of Obstetrics and Gynecology, Faculty of Medicine, University of Khartoum, 11111 Khartoum, Sudan; 3 Saad Abu Elella Teaching Hospital, University of Khartoum Fertility Centre, Khartoum, Sudan

**Keywords:** Infertility, Male, Female, Tubal factor, Ovulation, Sudan

## Abstract

**Background:**

Infertility is a big health problem worldwide. Few data exist on infertility in Sudan.

**Methods:**

A descriptive study was conducted to assess the pattern and the causes of infertility among couples (800) attending the University of Khartoum Fertility Centre, Saad Abualila Teaching Hospital in Khartoum, Sudan. The data on the socio-demographic characteristics of the patients, the type of infertility whether primary or secondary and the causes of infertility were extracted from the medical files retrospectively.

**Results:**

The mean (SD) age of the females was 32.4 (7.4) years while that of the males was 37.5 (7.2) years. The mean (SD) duration of infertility was 4.9(3.9) years. Five hundred and fifty one (68.9%) couples had primary infertility, while the remainder 249(31.1%) had secondary infertility. Two hundred and eighty four (35.5%) couples had male infertility, 342(42.8%) couples had female infertility. One hundred and forty seven (18.4%) couples had combined male and female infertility and in 27 (3.4%) couples the cause of infertility was not identified. Factors identified in the female infertility (342) were; anovulation (178, 52.05%), tubal factor (142, 41.52%), uterine factor (7, 2.05%) and other/combined (7, 2.05%). Azoospermia (75, 26.41%), oligozoospermia (45, 15.85%), asthenozoospermia (51, 17.96%), teratospermia (15, 5.28%) and mixed pathology (101, 35.56%) were the causes of the male infertility (*n* = 284). Female factors of infertility were observed more frequently among couples with secondary infertility compared with primary infertility (143/551(57.4) vs. 199/249(36.1), *P* < 0.001.

**Conclusion:**

The current study showed a high rate of primary infertility and female factor predominates compared with male factors. Future research direction should focus on the reasons why majority of clients seek this service very late.

## Background

Infertility is a big health problem worldwide as it has been estimated that in 2010 there are 48.5 million (45.0 million, 52.6 million) infertile couples worldwide [1]. World Health Organization (WHO) defines infertility as “a disease of the reproductive system defined by the failure to achieve a clinical pregnancy after 12 months or more of regular unprotected sexual intercourse” [2]. Meanwhile the WHO’s epidemiologic definition of infertility as “women of reproductive age at risk of becoming pregnant who report unsuccessfully trying for a pregnancy for more than two years” [3].

Among the various regions in the world, South East Asia and Sub-Saharan Africa countries have the highest prevalence of infertility [1]. Different causes and types of infertility were reported in the different African countries [4–7]. Investigating the types and causes of infertility is highly needed to generate data important for planning and interventions. While there is much data of infertility in high-income countries as well as in other African countries [1, 4–6, 8–11], few data exist on infertility in Sudan [12].

Sudan is the third largest country in Africa with a population of 36,787,000. There is a lack of proper infertility statistic in Sudan. However, infertility rate of 11.5% has been reported in 10 out of 18 Sudanese states [13]. While there is no governmental centre for assisted reproductive technology in Sudan, there are 10 private assisted reproductive technology centres in the capital Khartoum [14]. The current study was conducted to investigate the types and causes of infertility in University of Khartoum Fertility Centre, Saad Abualila Teaching Hospital in Khartoum, Sudan.

## Methods

A descriptive study was conducted at University of Khartoum Fertility Centre, Saad Abualila Teaching Hospital (Khartoum, Sudan). Consecutive infertile couples referred to the centre for assisted reproduction during the period of January – December 2017 were investigated thoroughly to identify the different causes of infertility. As mentioned above the WHO definition of infertility “failure to achieve a clinical pregnancy after 12 months or more of regular unprotected sexual intercourse” was followed [2]. The medical files of infertile couples were reviewed and the data were retrieved. Information which was extracted from the records included age, type of infertility, duration, factors identified as being responsible for infertility.

At University of Khartoum Fertility Centre, Saad Abualila Teaching Hospital the couples were assessed with a comprehensive history was taken, clinical examinations, gynecologic examination, trans-vaginal ultrasonography, hematological, hormonal profile (early follicular phase follicle stimulating hormone/luteinizing hormone or antimullerian hormone in those who are not cycling, thyroid stimulating hormone, dihyroepiandosterone sulphate), hysterosalpingography (laparoscopy was performed if it was indicated). Male factor of infertility was assessed by two semen analyses three months apart. Then the factor(s) responsible for the infertility were identified and recorded. Male factors and sperm parameters were interpreted according the WHO reference values [15]. The most common parameters of male infertility were; low sperm concentration (oligospermia), poor sperm motility (asthenospermia), and abnormal sperm morphology (teratospermia) [16].

Women were diagnosed as having polycystic ovarian syndrome (PCOS) according to Rotterdam criteria [17], which was based on fulfilling two more of the following criteria: “oligomenorrhoea/anovulation, clinical or biochemical hirsutism and morphology of polycystic ovaries on ultrasonography (≥12 follicles in each ovary measuring 2 to 9 mm in diameter” [17].

Women were diagnosed as having premature ovarian failure when there was no menarche (primary amenorrhea) or premature depletion of ovarian follicles/ arrested folliculogenisis (secondary amenorrhea) before the age of 40 years [18].

### Statistics

Data were entered in computer using SPSS for data analyses and expressed as proportions or mean (SD). *X* ^2^ was used to compare the proportions between the two groups. *P* value < 0.05 at two sided was considered statistically significant.

## Results

During the study period, records of 800 consecutive couples who attended the centre were reviewed. The range, mean (SD) age of the females was 16–46, 32.4 (7.4) years while that of the males was 14–60, 37.5 (7.2) years. The range, mean (SD) of the duration of infertility was 1–21, 4.9(3.9) years.

Five hundred and fifty one (68.9%) couples had primary infertility, while the remainder 249(31.1%) had secondary infertility.

**Fig. 1:**
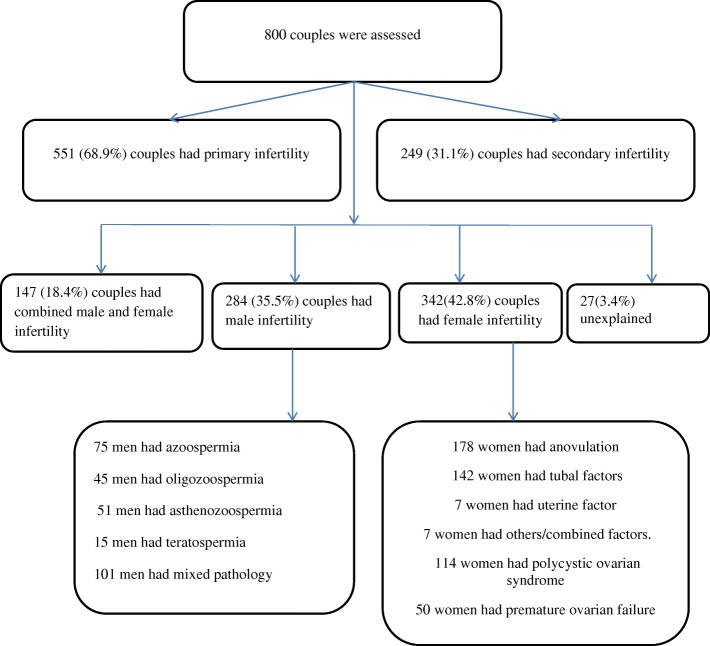
the flow chart and the causes of infertility in Khartoum, Sudan

Two hundred and eighty four (35.5%) couples had male factor only, 342(42.8%) couples had female factor only for infertility. One hundred and forty seven (18.4%) couples had combined male and female infertility and in 27 (3.4%) couples the cause of infertility was not identified, Fig. 1.

Factors identified in the female infertility (342) were; anovulation (178, 52.05%), tubal factor (142, 41.52%), uterine factor (7, 2.05%) and other/combined (7, 2.05%), Table 1. There were 164/800 (20.5%) women with PCO and 72/800 (9.0%) women had premature ovarian failure, Fig. 1.

**Table 1:** causes of female infertility in Khartoum Sudan (*n* = 342)

Variables	Number	proportion
Anovulation	178	52.05
Tubal factor	142	41.52
Uterine factor	7	2.05
Polycystic ovarian syndrome	114	33.33
Premature ovarian failure	50	14.62
Other/combined factors	7	2.05

Azoospermia (75; 26.41%), oligozoospermia (45, 15.85%), asthenozoospermia (51, 17.96%), teratospermia (15, 5.28%) and mixed pathology (101, 35.56%) were the causes of the male infertility (*n* = 284), Table 2.

**Table 2:** Causes of male infertility in Khartoum Sudan (n = 284)

Variables	Number	Proportion
Azoospermia	75	26.41
Oligozoospermia	45	15.85
Asthenozoospermia	51	17.96
Teratospermia	15	5.28
Mixed pathology	101	35.56

Compared with primary infertility, there was significantly a higher number of female factors of infertility among couples with secondary infertility (143/551(57.4) vs. 199/249(36.1), *P* < 0.001, Table 3.

**Table 3:** Comparing number (%) of the causes of infertility between primary and secondary infertility

Factors	Primary infertility (*n* = 551)	Secondary infertility (*n* = 249)	P
Male factors	225 (40.8)	59 (23.7)	< 0.001
Female factors	199 (36.1)	143 (57.4)	< 0.001
Combined infertility	114 (20.7)	33 (13.3)	0.011
Unexplained infertility	13 (2.4)	14 (5.6)	< 0.001

## Discussion

The main findings of the current study were the high rate of primary infertility (68.9%) and high rate of female factors among infertile couples. The high rate of primary infertility in this study was similar to our previous results in Khartoum where 443 (62.4%) couples had primary infertility and 267 (37.6%) couples had secondary infertility [12]. However, this is contrary to what have been reported in Nigeria [7, 10] and in Tanzania [8] where the secondary infertility (62.9%) predominated. In middle income countries e.g. Brazil in which over half (56.3%) of couples referred for in-vitro fertilization centre have secondary infertility [19]. Previous studies from developed countries have shown lower rates of secondary infertility [20]. The plausible explanation for the high rate of secondary infertility is high rate (85%) of tubal factor in Sub-Sahara Africa compared to lower rate (33%) in infertile women worldwide [20].

In the current study 284(35.5%) couples had male infertility, 342(42.8%) couples had female infertility, 18.4% of the couples had combined male and female infertility and in 3.4% couples the cause of infertility was not identified. This goes with the findings of Ugwu et al., who reported that the combined male/female factors were observed in 20.4% of cases in Nigeria [7]. A previous study in Benin documented that 32.2% of the couples have combined male and female factors [11]. However, other studies have reported that the causes of infertility may be associated with male factors (40%), female factors (40%) or the combination (20%) of both [4, 5]. Previous report have shown that tubal damage, male factor, anovulation and uterine factors as the main causes of infertility in Ghana [6]. Interestingly in Rwanda Dhont et al., [9] -who investigated 312 infertile women and their partners- have observed a low rate (3%) of unexplained infertility while half (50%) of the couples have combination of male and female causes. Likewise in Tanzania, Larsen et al., [8] have shown that female only factor was the cause of infertility in 65.9% of the couples, male only factor was identified in 6.8% of the couple and 15.2 and 12.1% of the couples had combined and unexplained infertility, respectively. A similar to low rate of unexplained infertility in our study (3.4%), a low rate of unexplained infertility was also reported in Rwanda (3%) [9] and in Nigeria (1.2%) [10]. It is worth mentioning that recent evidence-based meta-analysis and systematic review results have shown a decreased sperm concentration in the African male over past 50 years [21]. Multiple factors such as infectious, environmental, genetic, and dietary factors might have a role in causation of infertility [22].

Our results have shown that mean (SD) age of the female was 32.4 (7.4) years while that of the male was 37.5 (7.2) years. This is lower than the mean age of the women [34.1(4.9) year) which was previously reported in Nigeria [7]. Moreover, previous studies from Nigeria and India have reported the average age of the females and their husbands as 36.7/43.7 and 35/40 years, respectively [11, 23]. Perhaps couples have spent too much time treating infertility using conventional methods before presented to specialised centre. It is worth to be mentioned that in study in non-specialized centre in Benin Teaching Hospital, Nigeria, the mean age of women was 25 (3) years [24], however women presented at much higher (36.7 years) to IVF unit [25]. On the other hand the other reasons for presenting at advanced age include delayed marriage due to education and/or economic factors.

Our results documented the predominance of anovulation, while in most of the African countries tubal factor was the main of the cause of the female infertility [6–9]. The tubal factor could be due to infectious diseases such as *Neisseria gonorrhoeae* and *Chlamydia trachomatis* [8].

This was retrospective study and hospital-based one. Moreover, some socio-demographic variables e.g. history of smoking, alcohol intake were not available for analysis.

## Conclusion

The current study showed a high rate of primary infertility and female factor predominate. Future research direction should focus on the reasons why majority of clients seek this service very late.

## Data Availability

The datasets used and/or analysed during the current study are available from the corresponding author on reasonable request.

## Abbreviations

SD: Standard deviation
WHO: World Health Organization
